# New Insights on the Progesterone (P4) and PGRMC1/NENF Complex Interactions in Colorectal Cancer Progression

**DOI:** 10.3390/cancers15205074

**Published:** 2023-10-20

**Authors:** Joanna Kamińska, Olga Martyna Koper-Lenkiewicz, Donata Ponikwicka-Tyszko, Weronika Lebiedzińska, Ewelina Palak, Maria Sztachelska, Piotr Bernaczyk, Justyna Dorf, Katarzyna Guzińska-Ustymowicz, Konrad Zaręba, Sławomir Wołczyński, Nafis Ahmed Rahman, Violetta Dymicka-Piekarska

**Affiliations:** 1 Department of Clinical Laboratory Diagnostics, Medical University of Bialystok, Waszyngtona 15A, 15-269 Bialystok, Poland; o.koper@wp.pl (O.M.K.-L.); justyna.dorf@umb.edu.pl (J.D.); 2 Department of Biology and Pathology of Human Reproduction, Institute of Animal Reproduction and Food Research, Polish Academy of Sciences, 10-748 Olsztyn, Poland; d.ponikwicka-tyszko@pan.olsztyn.pl (D.P.-T.); e.palak@pan.olsztyn.pl (E.P.); maria.sztachelska@gmail.com (M.S.); 3 Department of Reproduction and Gynecological Endocrinology, Medical University of Bialystok, 15-269 Bialystok, Poland; weronika.lebiedzinska@umb.edu.pl (W.L.); slawek.wolczynski@gmail.com (S.W.); 4 Department of Medical Pathomorphology, Medical University of Bialystok, 15-269 Bialystok, Poland; piotr.bernaczyk@umwb.edu.pl; 5 Department of General Pathomorphology, Medical University of Bialystok, 15-269 Bialystok, Poland; katarzyna.guzinska-ustymowicz@umb.edu.pl; 6 2nd Clinical Department of General and Gastroenterological Surgery, Medical University of Bialystok, 15-094 Bialystok, Poland; konrad.zareba@umb.edu.pl; 7 Institute of Biomedicine, University of Turku, 20014 Turku, Finland; nafis.rahman@utu.fi

**Keywords:** biomarkers, colorectal cancer, neudesin (NENF), progesterone (P4), progesterone receptor membrane components 1 (PGRMC1)

## Abstract

**Simple Summary:**

Progesterone (P4) via PGRMC1/NENF may stimulate the proliferation and invasion of colorectal cancer DLD-1 and HT-29 cells. PGRMC1 inhibition abolishes the effect of P4, suggesting that P4 in advanced colorectal cancer may act primarily through PGRMC1. Our data may provide the novel insights into the action of P4, PGRMC1, and NENF in colorectal cancer. It seems that PGRMC1 and NENF may interact as possible cofactors in non-classical P4 signaling. Targeting the PGRMC1/NENF complex may open-up new therapeutic possibilities for patients with advanced colorectal cancer. Therefore, future studies aimed at developing treatment strategies for colorectal cancer could consider simultaneous PGRMC1 inhibition along with a blockage of NENF production and secretion.

**Abstract:**

The literature data regarding the risk of colorectal cancer (CRC) in the context of hormone therapy (HT), including both estrogen–progestogen combinations and estrogen alone, are inconclusive. The precise relationship underlying the action of progesterone (P4) and progesterone receptors in CRC has yet to be determined. We characterized the expression profiles of both nuclear and membrane progesterone receptors and their potential cofactors in CRC tissues. Additionally, we analyzed the P4 and NENF treatment effects on the cell proliferation and invasion of DLD-1 and HT-29 colorectal cancer cells. We observed a weak expression of the nuclear P4 receptor (PGR), but an abundant expression of the P4 receptor membrane component 1 (PGRMC1) and neuron-derived neurotrophic factor (NENF) in the CRC tissues. P4 treatment stimulated the proliferation of the DLD-1 and HT-29 CRC cells. The co-treatment of P4 and NENF significantly increased the invasiveness of the DLD-1 and HT-29 cells. A functional analysis revealed that these effects were dependent on PGRMC1. AN immunocytochemical analysis demonstrated a cytoplasmic co-localization of PGRMC1 and NENF in the CRC cells. Moreover, the concentration of serum NENF was significantly higher in CRC patients, and P4 treatment significantly increased the release of NENF in the DLD-1 cells. P4 or NENF treatment also significantly increased the IL-8 release in the DLD-1 cells. Our data may provide novel insights into the action of P4 and PGRMC1/NENF in CRC progression, where NENF may act as a potential PGRMC1 co-activator in non-classical P4 signaling. Furthermore, NENF, as a secreted protein, potentially could serve as a promising circulating biomarker candidate for distinguishing between colorectal cancer patients and healthy individuals, although large-scale extensive studies are needed to establish this.

## 1. Introduction

Colorectal cancer (CRC) is the third most common cancer worldwide, at an advanced stage with a 25% higher incidence rate in males than females [1,2,3]. Due to recurrence and distant metastasis [2], the mortality rate for colorectal cancer patients is very high [2,3], and tends to 30% and 40% in females and males, respectively [1]. Therefore, there is still a need to better investigate potential biomarkers for CRC diagnosis, as well as for the evaluation of the disease advancement, prognosis, and choice of rational therapeutic targets for personalized cancer treatment [4].

Progesterone (P4), an endogenous 21-carbon steroid hormone synthesized from cholesterol, is mainly produced by the corpus luteum and by the placenta during pregnancy. To a lesser extent, progesterone is also produced by the adrenal cortex, Leydig cells of the testes in men, adipose, and other tissues [5]. In addition to its reproductive importance in females, progesterone acts through multiple pathways, regulating important processes, e.g., brain development in fetuses, neuroprotection and myelin regeneration, immune response, and the proliferation and migration of various cancer cells in both genders [3,6,7]. P4 signals may be mediated by classical genomic or non-genomic action [7]. The classical P4 effect is dependent on the P4 interaction with the specific nuclear progesterone receptor (PGR) [8]. Rapid non-classical signaling is mediated by membrane P4 receptors (mPRα, mPRβ, mPRγ, mPRδ, and mPRε) and membrane-associated P4 receptors (MAPR), progesterone receptor membrane components 1 and 2 (PGRMC1 and PGRMC2) [9,10,11]. Among other MAPR proteins, important ones include neudesin (NENF, neuron-derived neurotrophic factor) and neuferricin (CYB5D2) [12]. *NENF* expression has been demonstrated in neurons and various peripheral tissues, such as the lungs, kidneys, and heart. NENF potentially promotes neuronal survival and differentiation by activating the MAPK (mitogen-activated protein kinase) and PI3K/AKT (phosphatidylinositol 3-kinase/protein kinase) pathways [13]. However, the distinct role of NENF in peripheral tissues remains unclear [14]. Recently, NENF has also been investigated as a molecule involved in the tumorigenesis of primary breast tumors, as well as in other human carcinomas of the uterine cervix, malignant lymphoma, colon, lung, skin, liver, and leukemia [15,16,17]. Our previous study showed elevated concentrations of NENF in the cerebrospinal fluid of patients with astrocytic brain tumors compared to non-tumoral controls, suggesting NENF as a circulating biomarker for brain tumors [17].

It is widely known that P4 plays a pivotal role in the development of breast, ovarian, and brain cancer [18]. Recent studies have suggested that steroid hormones may also affect CRC development, prognosis, and treatment [2]. Due to the increased morbidity and mortality rates for CRC and lack of specific CRC biomarkers [4], it seems crucial to identify the molecular mechanisms that promote CRC growth and metastasis. In recent years, targeted therapies for CRC seem to be promising treatment options [19]. Previous studies have confirmed the effectiveness of anti-progesterone receptor drugs in the treatment of breast, ovarian, lung, and head and neck cancers [20,21,22]. However, the mechanism of P4 action on progesterone receptors in CRC has not been well studied [2,3,23,24,25,26,27,28]. In the present study, we characterized the expression profiles of the nuclear and membrane P4 receptors and their cofactors in advanced colorectal cancers and investigated the potential molecular mechanism underlying the P4 action on CRC cell tissues and cell lines. Moreover, we evaluated NENF as a potential CRC biomarker.

## 2. Materials and Methods

### 2.1. Human Samples

All the samples were obtained from patients with primary colorectal cancer, who underwent surgical treatment at the 2nd Clinical Department of General and Gastroenterological Surgery of the Medical Clinical Hospital in Bialystok, Poland. Tissue samples for immunohistochemistry (IHC) were preserved in 4% formalin and, for a gene expression analysis, were preserved in snap frozen and stored at −80 °C. Human CRC tissues (*n* = 20; 14 males, 6 females, median age 68) and normal mucosa tissues (*n* = 10; 6 males, 4 females, median age 66) were histologically examined to prove the tumor grade at the Department of Medical Pathomorphology of the Medical University of Bialystok, Bialystok, Poland. Based on their symptoms, medical history, radiological, colonoscopy, and histological examination results, the CRC patients were retrospectively included. Histologically, CRC was classified as grade G2, intermediate grade (*n* = 8; 5 males, 3 females), and G3 high grade (*n* = 12; 9 males, 3 females). Blood samples from the CRC patients (*n* = 41, 27 males, 14 females, median age 69) were collected 1 day before surgery. The control group was composed of healthy volunteers, age- and sex-matched to the study group (*n* = 15; 11 males, 4 females, median age 67. The exclusion criteria encompassed other neoplasia and receiving chemo- or radiotherapy before surgery. 

### 2.2. Cell Cultures

Colorectal adenocarcinoma cell lines DLD-1 (CCL-221) and HT-29 (HTB-38), which differ in their resistance to anticancer treatment, were purchased from American Type Culture Collection, ATCC (Rockville, MD, USA). The DLD-1 cells were cultured in RPMI medium (RPMI 1640 Medium, no phenol red, Gibco™, catalog #: 11835030, Life Technologies Corporation, Grand Island, NE, USA) and the HT-29 cells were cultured in McCoy medium (McCoy’s 5A (Modified) Medium, GlutaMAX™ Supplement, Gibco™, catalog #: 36600021, Life Technologies Ltd., Paisley, UK), supplemented with 10% fetal bovine serum (FBS; Biochrom, Berlin, Germany), 100 units/mL of penicillin, and 100 µg/mL of streptomycin (P/S solution; Sigma-Aldrich) at 37 °C in a humidified atmosphere in the presence of 5% CO_2_. The cells were treated with P4 (1 µM) and AG-205 (1 µM). The dose of AG-205 was determined based on our previous studies [29,30] and NENF (1 ng/mL) in stimulation medium. Three independent experiments per cell line were run, and each performed cell plating was performed in triplicates for RNA isolation and medium collection. 

### 2.3. Drugs and Inhibitors

Progesterone (P4) and PGRMC1 inhibitor (AG-205) were obtained from Sigma-Aldrich (Saint Louis, MO, USA; catalog #: P8783-25G and A1487, respectively). Recombinant human Neudesin (NENF) was obtained from R&D Systems Europe Ltd., (Abingdon, UK; catalog #: 6714-ND-050).

### 2.4. Immunohistochemical Staining

The human CRC tissues and NM tissues were fixed in paraformaldehyde and embedded in paraffin. Immunohistochemical staining was carried out manually, as previously described [29,30]. Histological assessments were performed on 5 µm thick hematoxylin-eosin-stained sections. For immunohistochemistry, sections were deparaffinized, hydrated, and boiled in 10 mM of citric acid buffer (pH 6.0) in a retriever for 2.5 h. Tissue sections were incubated with blocking solutions (10% normal goat serum (NGS) with 3% bovine serum albumin (BSA) or only 3% BSA in PBS) for 1 h at room temperature to reduce non-specific background staining. Then, sections were incubated overnight at 4 °C with the primary antibodies for PGR (MA5-12658, Thermo Fisher Scientific Inc., Waltham, MA, USA; dilution 1:700), mPRα (ab75508, Abcam, Cambridge, UK; dilution 1:500), mPRβ (ab46534, Abcam; dilution 1:500), mPRγ (ab79517, Abcam; Cambridge, UK; dilution 1:500), PGRMC1 (PAB20135, Abnova Corporation, Taipei, Taiwan; dilution 1:300), PGRMC2 (ab125122, Abcam; Cambridge, UK; dilution 1:500), SERBP1 (ab28481, Abcam; Cambridge, UK; dilution 1:700), NENF (MAB6714, R&D Systems Europe Ltd. Abingdon, UK; dilution 25 µg), IgG (ab190475, Abcam; Cambridge, UK; dilution 1:700), and IgG2a (ab190463, Abcam; Cambridge, UK; dilution 1:500). After endogenous peroxidase blocking (0.5% H_2_O_2_ in PBS for 20 min in dark at room temperature), the primary antibodies were linked with Envision^®^ anti-mouse or anti-rabbit polymer + HRP (Dako, Glostrup, Denmark) for 30 min at room temperature. The reaction product was visualized using 3′3-diaminobenzidine tetrahydrochloride (DAB, Dako, Glostrup, Denmark). Each step was followed by three washings using PBS with 0.05% Tween 20 (PBS-T). After staining with hematoxylin, the sections were dehydrated through ascending ethanol concentrations and cleared using xylene. They were then mounted with Pertex (Histolab Products AB, Spånga, Sweden).

### 2.5. ImageJ Analysis

The intensity of the staining was determined by measuring the optical density of the reaction product, which was analyzed using Fiji Software 1.8.0_172 (Fiji Is Just ImageJ). Six random areas from each section were quantified and the average optical density (OD) was calculated for each of these areas.

### 2.6. Immunocytochemical Staining

To minimize autofluorescence in dual staining, tissues were treated with 100 mM of NH4Cl for 10 min. The blocking solution, a mixture of 5% NGS and 1% BSA in PBST, was then applied for 1 h at room temperature. After blocking unspecific binding sites with 3% BSA in PBS with 0.05% Tween 20 for 30 min, the tissue slides were incubated with primary antibodies for PGRMC1 (PAB20135 from Abnova Corporation, Taipei, Taiwan; dilution 1:300) and NENF (MAB6714 from R&D Systems Europe Ltd., Abingdon, UK; dilution 25 µg) diluted in the blocking solution for 1 h. Following the previous step, the tissue slides were incubated in the dark with the secondary fluorescent antibodies Alexa Fluor 594 and 488 goat anti-mouse (ab150116 from Abcam, Cambridge, UK; dilution 1:500 and ab150113 from Abcam, Cambridge, UK; dilution 1:500, respectively) for 1 h. Cell nuclei were detected by incubating the tissue slides with DAPI.

### 2.7. RNA Isolation

The total RNA was isolated from the colorectal cancer and NM tissues, DLD-1, and HT-29 cell lines using the TRIzol-based extraction method (Invitrogen, Carlsbad, CA, USA; catalog #: 15596018). The quantity and quality of the extracted RNA were assessed by measuring its absorbance using the Synergy HTX Multi-Mode Reader (Agilent, Santa Clara, CA, USA). The integrity of the isolated RNA was confirmed by performing gel electrophoresis.

### 2.8. Real-Time RT-PCR

Before the reverse transcription (RT) reaction, 1 µg of the total RNA was treated with DNase I, Amplification Grade (Invitrogen, Carlsbad, CA, USA; catalog number 18068-015) following the manufacturer’s instructions. The RT reaction was carried out with the SensiFAST cDNA Synthesis Kit (Bioline Reagents Ltd., London, UK; catalog #: BIO-65054), according to the manufacturer’s protocol. The expressions of the target genes were quantified using the StepOnePlusTM Real-Time PCR System (Applied Biosystems™, Thermo Fisher Scientific, Life Sciences Solutions Group, Carlsbad, CA, USA) and Power SYBR™ Green PCR Master Mix (Applied Biosystems™, catalog #: 4368706, Thermo Fisher Scientific Baltics UAB, Vilnius, Lithuania).

The reaction conditions were as follows: an initial denaturation step at 95 °C for 10 min, followed by 40 cycles of amplification at 95 °C for 15 s, 56–60 °C for 45 s, and 70 °C for 45 s. A melting curve analysis was performed at the end of the PCR reaction to verify that only a single product was amplified. The amplification products were separated on 1.5% agarose gel and stained with ethidium bromide. The expression levels were normalized to the housekeeping gene peptidylprolyl isomerase A (*PPIA*). The sequences of the primers and the expected product sizes are listed in Supplementary Table S2. Each reaction product was verified using a sequencing analysis.

### 2.9. Cell Proliferation

The proliferation of the DLD-1 and HT-29 cell lines was assessed using two methods after being treated for 24, 48, and 72 h. The first method was the CellTiter 96^®^ AQueous Non-Radioactive Cell Proliferation Assay (Promega, Madison, WI, USA, catalog #: G4000) and the second was the BrdU Cell Proliferation Assay Kit (Cell Signaling Technology, Danvers, MA, USA, catalog #: 6813). The medium containing the drugs was changed every 24 h, while the control groups were treated with a starvation medium (RPMI/McCoy’s medium with 0.5% FBS and P/S solution). The metabolic activity of living cells was measured using the MTT (3-(4,5-dimethylthiazol-2-yl)-2,5-diphenyltetrazolium bromide) assay, which evaluates the conversion of a tetrazolium salt into a formazan product. The cells were subjected to the tetrazolium salt for 4 h and the measurement was performed using spectrophotometry. The BrdU assay evaluated the incorporation of 5-bromo-2′-deoxyuridine (BrdU) into the DNA of the cells that were exposed to 10 μM of the substance for 12 h. The cells were then fixed and treated with an anti-BrdU antibody, and the magnitude of the absorbance was used to assess the incorporation of BrdU into the DNA. The results were read using a plate reader Infinite M200 Pro (Tecan Trading AG, Männedorf, Switzerland) and are presented as a percentage of the control group, which was set at 100%. Each experiment was run three times with eight replicates.

### 2.10. Cell Invasion

The invasion intensity of the DLD-1 and HT-29 cells was determined using the CultreCoat^®^ 96 Well Medium BME Cell Invasion Assay from R&D Systems (catalog #: 3482-096-K). In brief, 2.5 × 10^4^ cells were placed in each well of a 96-well plate, with the top chamber coated in Medium Basement Membrane Extract (BME). The invasion of the cells in response to P4 (1 µM) and AG-205 (1 µM) was measured using Calcein AM after 24 h of treatment. Free Calcein fluoresces brightly, and was used to quantify the number of cells that invaded or migrated in comparison to a standard curve. The invasion intensity of the treated groups was expressed as a percentage of the control group, which was set at 100%. The results were obtained from three separate experiments, each consisting of eight replicates.

### 2.11. ELISA Evaluation

The levels of NENF and IL-8 were analyzed using sandwich enzyme-linked immunosorbent assay (ELISA) kits. No dilution was performed on the samples before analysis, and the experiments were performed according to the manufacturer’s guidelines. The concentrations of NENF in the CRC patient serum were measured using the Human Neudesin ELISA Kit from EIAB Science Inc, Wuhan, China (catalog #: E13396h), with an intra-assay coefficient of variation (CV%) of ≤4.8% and an inter-assay CV% of ≤7.1%, according to the manufacturer. The levels of IL-8 in the cell lines’ medium were measured using the ELISA Quantikine^®^ Human IL-8/CXCL8 Immunoassay kit (catalog #: D8000C) from R&D Systems Europe Ltd., Abingdon, UK. The manufacturer reported an intra-assay CV% of 5.6% at an IL-8 mean concentration of 168 pg/mL.

### 2.12. Statistical Analysis

The obtained results were analyzed with the STATISTICA 13.0 PL software (StatSoft Inc., Tulsa, OK, USA) and the GraphPad Prism v.8.4.3 (GraphPad Software, Inc., San Diego, CA, USA). The results are expressed as mean ± SEM. The Mann–Whitney test was used to compare two independent samples. A receiver operator characteristic (ROC) curve was generated to calculate the area under the ROC curve (AUC). To indicate the optimal cut-off point (threshold value)m the Youden index was estimated. Differences were considered significant for a two-tailed *p* < 0.05 level and are denoted by an asterisk (* *p* ≤ 0.05, ** *p* ≤ 0.01, *** *p* ≤ 0.001, and **** *p* ≤ 0.0001).

## 3. Results

### 3.1. Nuclear and Membrane P4 Receptors Are Expressed in CRC Tissues and Cell Lines

**Figure 1:**
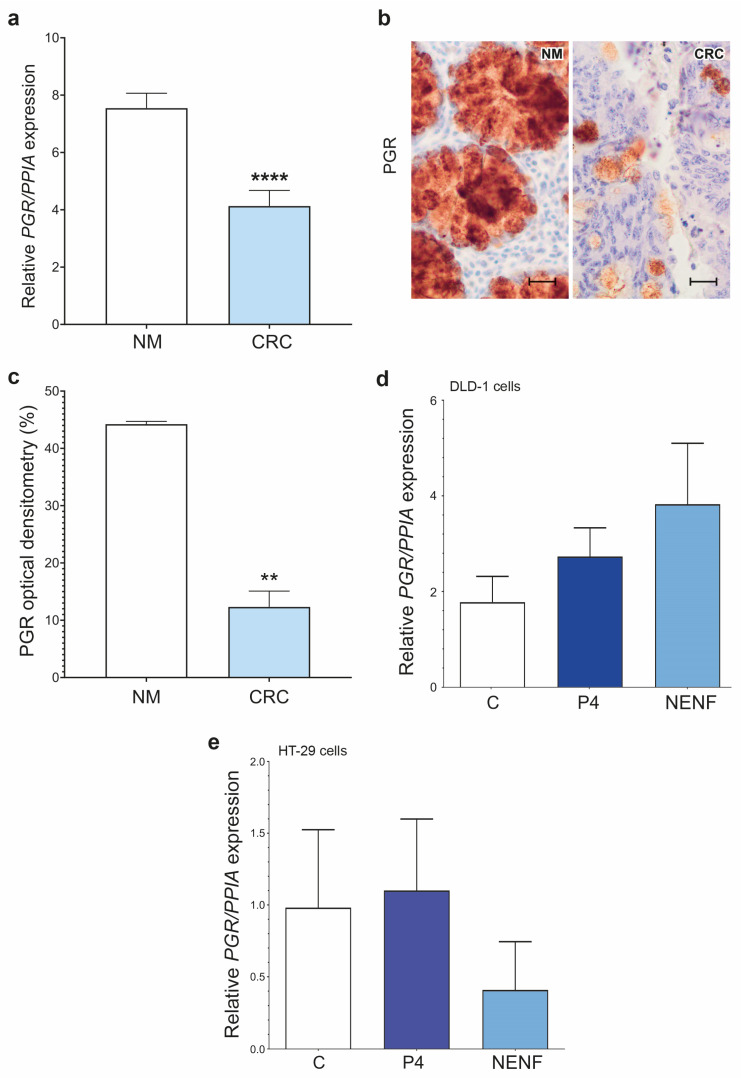
Characterization of *PGR* expression levels in colorectal cancer and DLD-1 and HT-29 cell lines. *PGR* expression at gene, (**a**) and protein (**b**,**c**) levels in NM (*n* = 10) and colorectal cancer (CRC) tissues (*n* = 20). Original magnification, 20×; scale bar, 20 µm. The columns represent the mean ± SEM relative to *PPIA*. The Mann–Whitney test was used to compare NM vs. CRC results. Statistical significance of NM vs. CRC: **** *p* ≤ 0.0001, ** *p* ≤ 0.01. *PGR* expression after treatment with 1 µM of P4 and 1 µg/mL of NENF in DLD-1 cell line (**d**) and HT-29 cell line (**e**) (*n* = 3 independent experiments). The Mann–Whitney test was used to compare C vs. P4 and C vs. NENF of DLD-1 and HT-29 cells. The differences are statistically non-significant. Abbreviations: C, control/non-treated group; CRC, colorectal cancer; NENF, neudesin; NM, normal mucosa; P4, progesterone; PGR, nuclear progesterone receptor; PPIA, peptidylprolyl isomerase A; SEM, standard error of the mean; and vs., versus.

**Figure 2:**
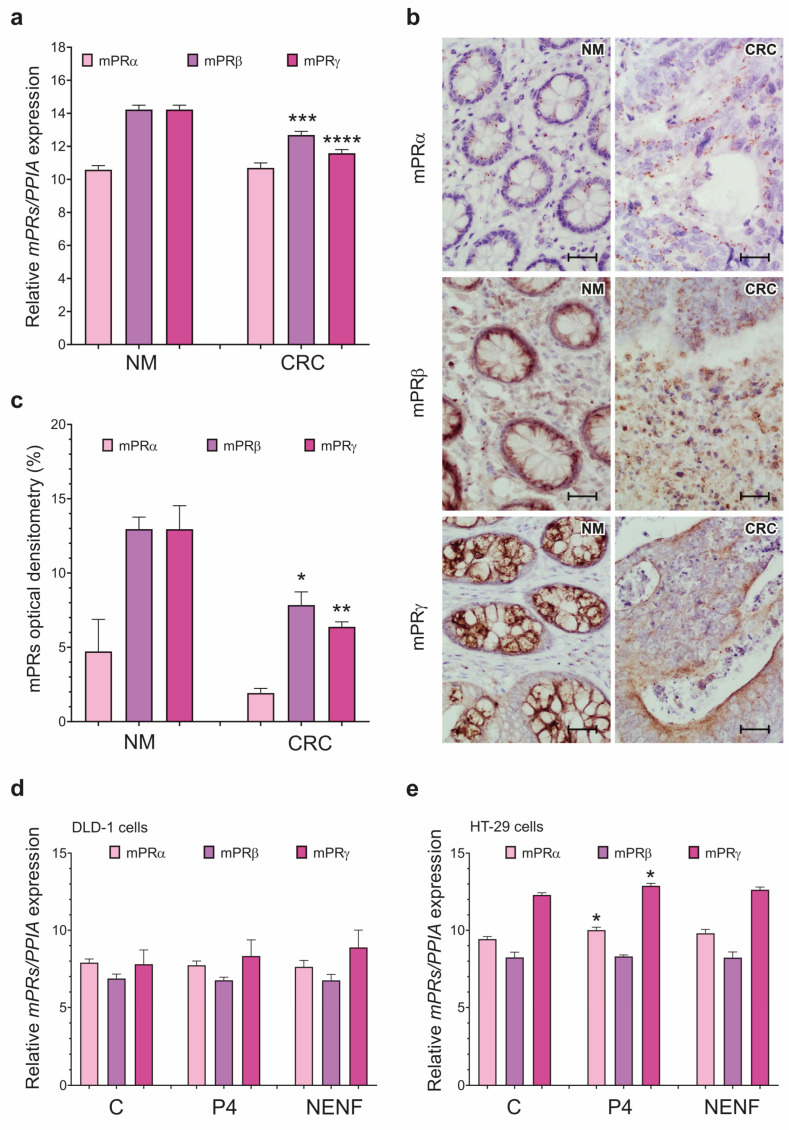
Characterization of *mPRα*, *mPRβ*, and *mPRγ* expression levels in colorectal cancer and DLD-1 and HT-29 cell lines. *mPRα*, *mPRβ*, and *mPRγ* expression at gene (**a**) and protein (**b**,**c**) levels in NM (*n* = 10) and colorectal cancer (CRC) tissues (*n* = 20). Original magnification, 20×; scale bar, 20 µm. The columns represent the mean ± SEM relative to *PPIA*. The Mann–Whitney test was used to compare NM vs. CRC results. Statistical significance of NM vs. CRC for *mPRβ*, *mPRγ*: * *p* ≤ 0.05, ** *p* ≤ 0.01, *** *p* ≤ 0.001, and **** *p* ≤ 0.0001. *mPRα*, *mPRβ*, and *mPRγ* expression after treatment with 1 µM of P4 and 1 µg/mL of NENF in DLD-1 cell line (**d**) and HT-29 cell line (**e**) (*n* = 3 independent experiments). The Mann–Whitney test was used to compare C vs. P4 and C vs. NENF. Statistical significance of C vs. P4 for *mPRα*, *mPRγ* of HT-29 cells: * *p* ≤ 0.05. Other differences are statistically non-significant. Abbreviations: C, control/non-treated group; CRC, colorectal cancer; mPRα, membrane progesterone receptor alfa; mPRβ, membrane progesterone receptor beta; mPRγ, membrane progesterone receptor gamma; NENF, neudesin; NM, normal mucosa; P4, progesterone; PPIA, peptidylprolyl isomerase A; SEM, standard error of the mean; and vs., versus.

**Figure 3:**
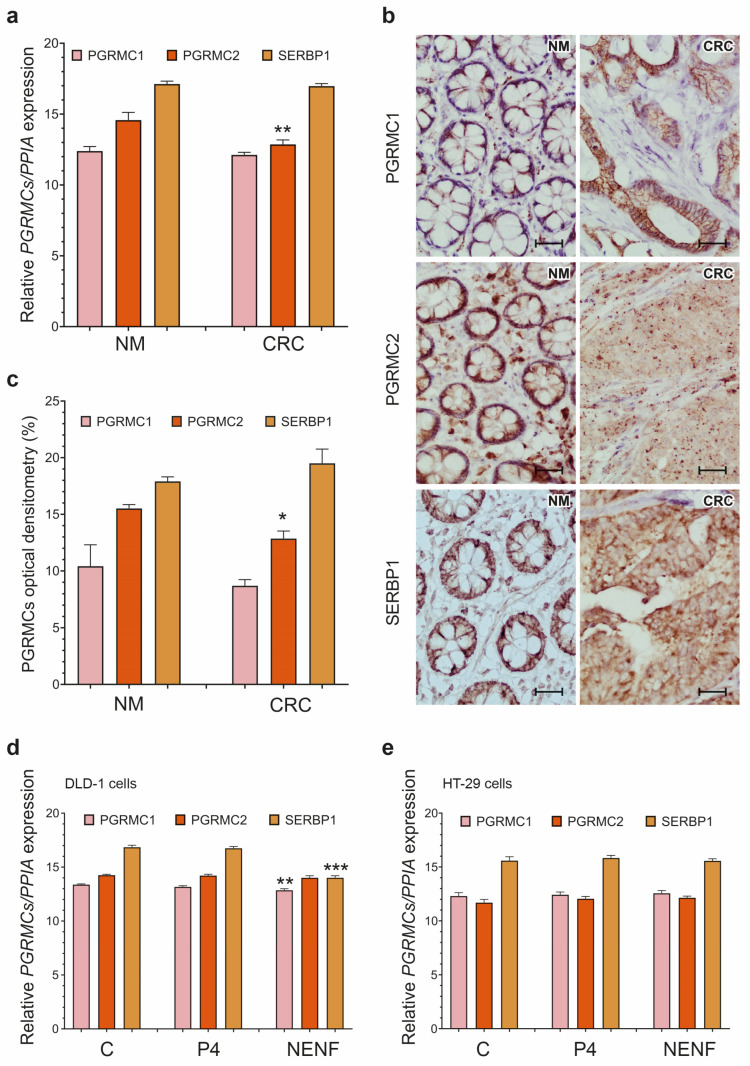
Characterization of *PGRMCs* expression levels in colorectal cancer and DLD-1 and HT-29 cell lines. *PGRMC1*, *PGRMC2*, and *SERBP1* expression at gene (**a**) and protein (**b**,**c**) levels in NM (*n* = 10) and colorectal cancer (CRC) tissues (*n* = 20). Original magnification, 20×; scale bar, 20 µm. The columns represent the mean ± SEM relative to *PPIA*. The Mann–Whitney test was used to compare NM vs. CRC results. Statistical significance of NM vs. CRC for *PGRMC2*: * *p* ≤ 0.05, ** *p* ≤ 0.01. Other differences are statistically non-significant. *PGRMC1*, *PGRMC2*, and *SERBP1* expression after treatment with 1 µM of P4 and 1 µg/mL of NENF in DLD-1 cell line (**d**) and HT-29 cell line (**e**) (*n* = 3 independent experiments. The Mann–Whitney test was used to compare C vs. P4 and C vs. NENF. Statistical significance of C vs. NENF for *PGRMC1* and *SERBP1* of DLD-1 cells: ** *p* ≤ 0.01, *** *p* ≤ 0.001. Other differences are statistically non-significant. Abbreviations: C, control/non-treated group; CRC, colorectal cancer; NENF, neudesin; NM, normal mucosa; P4, progesterone; PGRMC1, progesterone receptor membrane component 1; PGRMC2, progesterone receptor membrane component 2; PPIA, peptidylprolyl isomerase A; SEM, standard error of the mean; SERBP1, SERPINE1 mRNA binding protein; and vs., versus.

We screened the CRC tissues and DLD-1 and HT-29 cells for the expression profiling of all PR types (Figure 1, Figure 2 and Figure 3, Supplementary Figure S1). The expression of *PGR* was significantly down-regulated in the CRC compared to normal mucosa (NM) tissues (Figure 1a). The IHC analysis showed a weak nuclear PGR signal in the CRC tissues compared to an abundant expression in the glandular cells of the NM tissues (Figure 1b). Densitometric quantification and an optical density (OD) evaluation showed significantly decreased PGR protein expression in the CRC compared to NM tissues (Figure 1c). P4 or NENF treatment did not have any effect on the *PGR* expression level in both the DLD-1 and HT-29 cell lines (Figure 1d,e). 

The expression of *mPRα* was unchanged in the CRC and NM tissues, whereas *mPRβ* and *mPRγ* were significantly down-regulated in the colorectal cancer compared to NM tissues (Figure 2a). The IHC analysis showed a weak mPRα signal in both the CRC and NM tissues, and weak mPRβ and mPRγ cytoplasmic expressions in the CRC tissues (Figure 2b). The OD evaluation revealed significantly decreased mPRβ and mPRγ protein expressions in the CRC tissues compared to the NM tissues (Figure 2c). P4 or NENF treatment did not affect the *mPRs* in the DLD-1 cells (Figure 2d), whereas P4 significantly up-regulated *mPRα* and *mPRγ* expressions in the HT-29 cells (Figure 2e).

The expression of *PGRMC1* and its potential cofactor SERPINE 1 mRNA binding protein 1 (*SERBP1*) was similar in the CRC and NM tissues, whereas the gene expression of *PGRMC2* was significantly down-regulated in the CRC tissues (Figure 3a). Immunolocalization studies detected PGRMC1, PGRMC2, and SERBP1 expression in the cytoplasm of both the CRC and NM tissues. IHC showed abundant PGRMC1 and SERPINE expressions in the CRC tissues (Figure 3b). The OD evaluation revealed a significantly decreased PGRMC2 protein expression in the CRC tissues compared to the NM tissues (Figure 3c). NENF treatment significantly down-regulated the expressions of *PGRMC1* and *SERBP1* in the DLD-1 cells (Figure 3d), while P4 or NENF treatment did not have any effect on *PGRMC’s* expression in the HT-29 cells (Figure 3e).

### 3.2. NENF Level Is Upregulated in Colorectal Cancer

**Figure 4:**
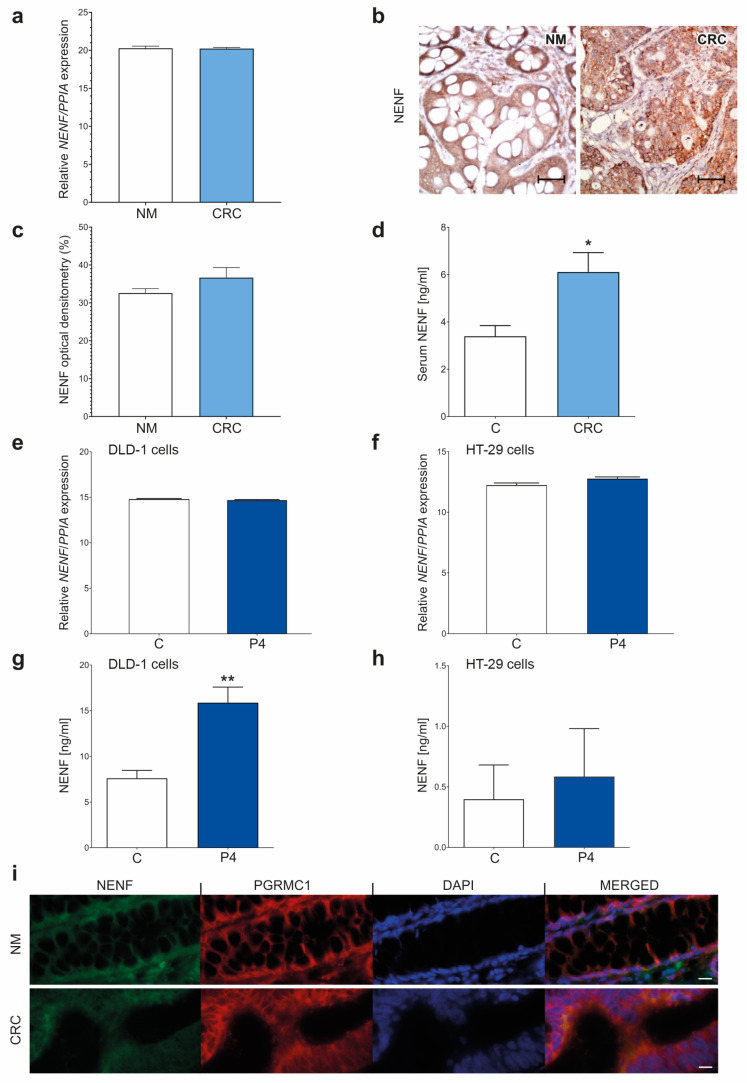
Characterization of *NENF* expression and release in colorectal cancer patients and DLD-1 and HT-29 cell lines. *NENF* expression at gene (**a**) and protein (**b**,**c**) levels in NM (*n* = 10) and colorectal cancer (CRC) tissues (*n* = 20). Original magnification, 20×; scale bar, 20 µm. The columns represent the mean ± SEM relative to *PPIA*. The Mann–Whitney test was used to compare NM vs. CRC results. The differences are statistically non-significant. Serum NENF concentration in colorectal cancer patients (*n* = 41) compared to the control group (*n* = 15) (**d**). The Mann–Whitney test was used to compare serum NENF concentration in C vs. CRC results. Statistical significance: * *p* ≤ 0.05. *NENF* expression after treatment with 1 µM of P4 in DLD-1 cell line (**e**) and HT-29 cell line (**f**) (*n* = 3 independent experiments). The Mann–Whitney test was used to compare *NENF* expression in C vs. CRC results in DLD-1 and HT-29 cells. The differences are statistically non-significant. NENF concentration in the medium after treatment with 1 µM of P4 in DLD-1 cell line (**g**) (*n* = 6) and HT-29 cell line (**h**) (*n* = 6). The Mann–Whitney test was used to compare NENF concentration in the medium C vs. P4 results of DLD-1 cells, statistical significance: ** *p* ≤ 0.01. Double staining for PGRMC1 and NENF in NM tissues (*n* = 10) and colorectal cancer (*n* = 20) (**i**). PGRMC1-positive cells are in red, NENF-positive cells are in green, nucleus localization in cells is in blue, and PGRMC1 and NENF merged staining is in orange, scale bar, 20 μm. Abbreviations: C, control/non-treated group; CRC, colorectal cancer; NENF, neudesin; NM, normal mucosa; P4, progesterone; PGRMC1, progesterone receptor membrane component 1; PPIA, peptidylprolyl isomerase A; SD, standard deviation; SEM, standard error of the mean; and vs., versus.

We assessed the *NENF* expression in colorectal cancer and its release in CRC tissues and DLD-1 and HT-29 cell lines. The mRNA of the *NENF* expression level was similar in the CRC and NM tissues (Figure 4a). IHC showed abundant NENF expression in the CRC tissues (Figure 4b). The OD evaluation indicated that the protein expression in the CRC tissues was comparable to that in the NM tissues (Figure 4c). However, the NENF concentration was significantly higher in the serum of the CRC patients compared to the healthy controls (Figure 4d). A receiver operator characteristic (ROC) curve analysis showed that the serum NENF score significantly differentiated the colorectal cancer patients from the healthy controls with a diagnostic sensitivity and positive predictive value of 83% and 81%, respectively (Supplementary Table S3, Supplementary Figure S2). P4 treatment did not affect the *NENF* in both the DLD-1 and HT-29 cells (Figure 4e,f), however, P4 treatment significantly increased the release of NENF in the DLD-1 cells (Figure 4g), but not in the HT-29 cells (Figure 4h). Immunocytochemical staining colocalized both PGRMC1 and NENF in the cytoplasm of the CRC and NM tissues (Figure 4i).

### 3.3. P4 Treatment Affects the DLD-1 and HT-29 Cell Proliferation, but in Combination with NENF Also Promotes Cell Invasion

**Figure 5:**
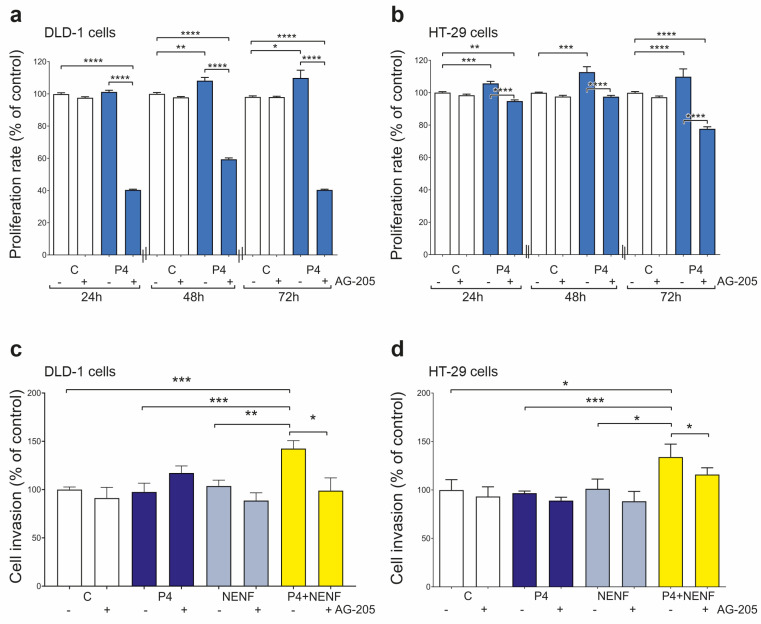
P4 and NENF treatment effect on cell proliferation and invasion in DLD-1 and HT-29 cell lines. Effects of 1 µM of P4 with or without 1 µM of PGRMC1 inhibitor (AG-205) on the proliferation rate of DLD-1 (**a**) and HT-29 (**b**) cell lines after 24 h, 48 h, and 72 h treatments (*n* = 3 independent experiments). The Mann–Whitney test was used to compare C vs. P4 results without or with 1 µM of PGRMC1 inhibitor (AG-205), and compare P4 without AG-205 vs. P4 with AG-205 of DLD-1 and HT-29 cells, statistical significance: * *p* ≤ 0.05, ** *p* ≤ 0.01, *** *p* ≤ 0.001, and **** *p* ≤ 0.0001. Other differences are statistically non-significant. Effects of 1 µM of P4 or/and 1 µg/mL of NENF treatments without or with 1 µM of AG-205 on cell migration of DLD-1 (**c**) and HT-29 (**d**) cell lines after 24 h treatment (*n* = 3 independent experiments). The Mann–Whitney test was HT-29 cells, statistical significance: * *p* ≤ 0.05, ** *p* ≤ 0.01, and *** *p* ≤ 0.001. Other differences are statistically non-significant. Cell proliferation and cell invasion rates of the treated and non-treated (control) groups are presented as the percentage of the control, considered as 100%. Abbreviations: AG-205, PGRMC1 inhibitor; C, control/non-treated group; NENF, neudesin; P4, progesterone; PGRMC1, progesterone receptor membrane component 1; and vs., versus. Used to compare C vs. P4 + NENF results without or with 1 µM of PGRMC1 inhibitor (AG-205); compare P4 vs. P4 + NENF without or with AG-205; and compare NENF vs. P4 + NENF without or with AG-205 of DLD-1 and.

We examined the effects of P4 and NENF on the cell proliferation and invasion of DLD-1 and HT-29 colorectal cancer. P4 significantly stimulated cell proliferation after 48 h and 72 h in the DLD-1 cell line, and after 24 h, 48 h, and 72 h in the HT-29 cell lines (Figure 5a,b). PGRMC1 blockage with the PGRMC1 inhibitor AG-205 inhibited the P4 effect in both cell lines (Figure 5a,b). P4 or NENF treatment alone did not affect the cell invasion of the DLD-1 and HT-29 cells (Figure 5c,d). However, P4 and NENF co-treatment significantly increased the cell invasion of the DLD-1 and HT-29 cells, and this effect could be abolished by AG-205 (Figure 5c,d).

### 3.4. P4 and NENF Up-Regulate IL-8 Expression and Its Release in DLD-1 and HT-29 Cells

**Figure 6:**
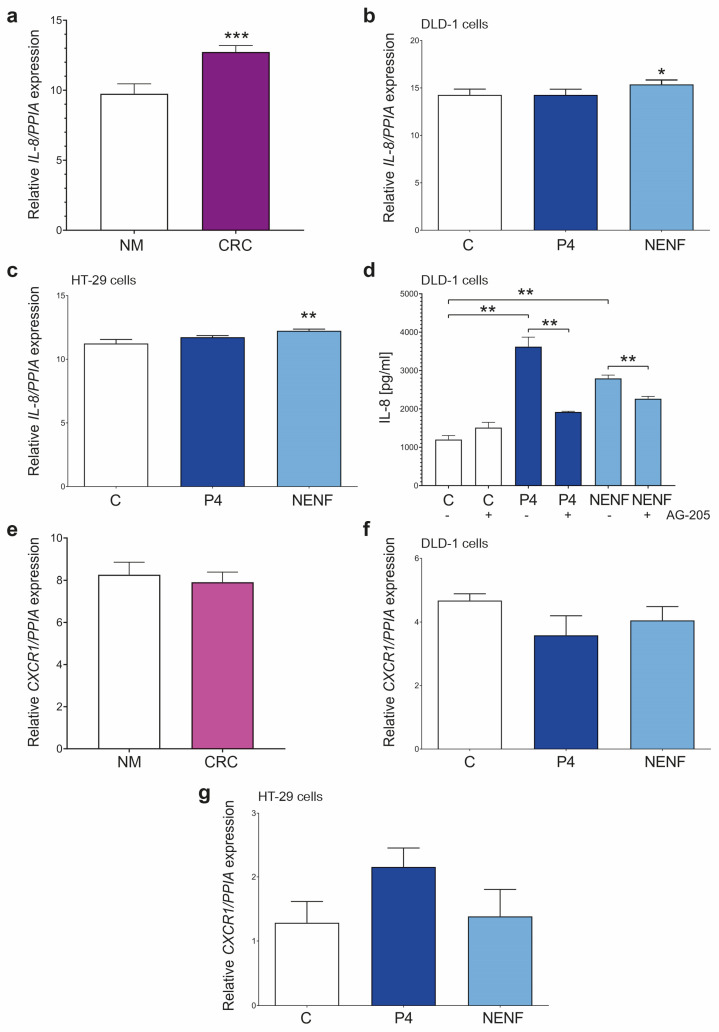
Characterization of *IL-8* and *CXCR1* expression and release in colorectal cancer patients and DLD-1 and HT-29 cell lines. *IL-8* expression in NM (*n* = 10) and colorectal cancer (*n* = 20) tissues (**a**). The columns represent the mean ± SEM relative to *PPIA*. The Mann–Whitney test was used to compare NM vs. CRC results, statistical significance of NM vs. CRC for *IL-8*: *** *p* ≤ 0.001. *IL-8* expression after treatment with 1 µM of P4 or with 1 µg/mL of NENF in DLD-1 (**b**) and HT-29 (**c**) cell lines (*n* = 3 independent experiments). The Mann–Whitney test was used to compare C vs. P4 and C vs. NENF results of DLD-1 and HT-29 cells, statistical significance of C vs. NENF: * *p* ≤ 0.05, ** *p* ≤ 0.01. IL-8 concentration in the medium of DLD-1 cell line after treatment with 1 µM of P4 or 1 ng/mL of NENF without or with 1 µM of AG-205 (**d**) (*n* = 6). The Mann–Whitney test was used to compare C vs. P4 without or with 1 µM of AG-205, C vs. NENF without or with 1 µM of AG-205, and also results of P4 without AG-205 vs. P4 with AG-205 of DLD-1 or NENF without AG-205 vs. NENF with AG-205 of DLD-1 cells, statistical significance: ** *p* ≤ 0.01. *CXCR1* expression in NM (*n* = 10) and colorectal cancer (*n* = 20) tissues (**e**). The Mann–Whitney test was used to compare NM vs. CRC results. The differences are statistically non-significant. *CXCR1* expression after treatment with 1 µM of P4 or with 1 µg/mL of NENF in DLD-1 (**f**) and HT-29 (**g**) cell lines (*n* = 3 independent experiments). The Mann–Whitney test was used to compare C vs. P4 and C vs. NENF results of DLD-1 and HT-29 cells. The differences are statistically non-significant. Abbreviations: AG-205, PGRMC1 inhibitor; C, control/non-treated group; CRC, colorectal cancer; CXCR1, C-X-C Motif Chemokine Receptor 1; IL-8, interleukin 8; NENF, neudesin; NM, normal mucosa; P4, progesterone; PGRMC1, progesterone receptor membrane component 1; PPIA, peptidylprolyl isomerase A; SEM, standard error of the mean; and vs., versus.

We assessed the expression of *IL-8* in the CRC tissues and checked the P4 and NENF treatment effect on the expression and release of IL-8 and its receptor CXCR1 in the DLD-1 and HT-29 cell lines. The expression of *IL-8* was significantly up-regulated in the CRC compared to the NM tissues (Figure 6a). NENF treatment significantly up-regulated *IL-8* in the DLD-1 and HT-29 cells (Figure 6b,c). P4 or NENF treatment significantly increased the IL-8 release in both the DLD-1 and HT-29 cells, whereas AG-205 significantly abolished this effect (Figure 6d). The expression of the IL-8 receptor *CXCR1* was unaffected in the CRC and NM tissues (Figure 6e). P4 or NENF treatment did not have *any effect on the *CXCR1* expression in both cell lines (Figure 6f,g).

## 4. Discussion

The available data in the medical literature on the risk of colorectal cancer (CRC) in the context of hormone therapy (HT) are still unconvincing. Recently, hormone therapy has been linked to a decreased risk of colorectal cancer (CRC) [31,32,33]. Lin et al. suggested that both estrogen–progestogen therapy (EPT) and estrogen therapy (ET), especially when used currently, are associated with a reduced risk of colorectal cancer in peri- or postmenopausal women. EPT demonstrates a more consistent association with the reduction in CRC risk, regardless of the duration of its use [34]. The use of hormone replacement therapy (HRT) has been connected to a significant reduction in the risk of both colorectal-cancer-specific mortality and all-cause mortality in women with colorectal cancer. The authors emphasized the hormone-dependent nature of CRC and its inverse association with tumor progression concerning estrogen receptor β (ERβ) expression [35]. However, the available literature also suggests that the use of HRT is not associated with an increased risk or even the possibility of CRC [36]. Clinical studies of the Women’s Health Initiative (WHI) have revealed an increased risk of breast cancer in women using HRT [37]. In the context of breast cancer, progesterone plays a complex role by influencing cell growth and division, regulating autocrine mechanisms, and interacting with other growth factors. It can impact the development of both receptor-positive (estrogen/progesterone receptor-positive) and receptor-negative tumors, making its role in carcinogenesis multifaceted [38,39]. The studies describing the relationship between HT and ovarian cancer also are inconclusive [40]. They have suggested both an increased risk of ovarian cancer with long-term use of HRT [41,42], and no significant difference in ovarian cancer incidence between a HRT group and a placebo group [43].

The expression of all P4 receptors in colorectal cancer has not been well-characterized, and the existing data in the available literature have been conflicting and inconclusive (Supplementary Table S1) [2,3,23,24,25,26,27,28]. This suggests that further research is needed to fully understand the role of P4 receptors in colorectal cancer to determine whether they could serve as potential therapeutic targets. In our study, we found that the mRNA and protein levels of PGR, mPRβ, mPRγ, and PGRMC2 were significantly down-regulated in the CRC compared to the normal tissues. A low expression of PGR has been associated with a poor prognosis of CRC [28]. The abundant expression of PGRMC1 in advanced stages of CRC suggests its potential role in cancer progression. Most studies have, to date, focused on the expression and role of PGRMC1 in cancers other than colorectal cancer, such as ovarian [44], breast [45], endometrial [46], lung [47], and hepatocellular carcinoma [48]. These studies have shown that PGRMC1 plays a role in promoting tumor growth and cell proliferation [49], anchorage-independent growth, migration, invasion [50], resistance to chemotherapy [44], tumor angiogenesis regulation, and cancer cell apoptosis suppression [47]. Our present observations on the P4 receptor expression pattern in CRC are consistent with the recently reported marginal expression of PGR, with low expression levels of mPRβ, mPRγ, and PGRMC2, and abundant expression levels of PGRMC1 in high-grade human ovarian cancer [29]. A significantly up-regulated expression of PGRMC1 in advanced human ovarian cancers has been suggested to indicate its important role in disease progression [51]. Moreover, elevated PGRMC1 expression in breast cancer has been linked to more advanced stages and a poor prognosis [52]. Our results also suggested that PGRMC1 may play a pivotal role in CRC progression, however, the molecular mechanism of PGRMC1 action in cancers is still not fully understood.

P4 treatment has been shown to inhibit the proliferation of various colorectal cancer cell lines by stopping the G2/M phase of the cell cycle and inducing apoptosis [28]. However, the P4 doses used in this study were supraphysiological and most likely clinically irrelevant, due to the very rapid metabolism of P4 [28]. The inhibitory effect of P4 has also been shown in vivo, but information on the dose used in the mice treatment is lacking [28]. However, SW620 cells with a higher expression of the PGR were used for inoculation, suggesting that P4 may have an inhibitory effect on CRC with a high expression of PGR [28]. In our study, we chose cell lines with a very low/traceable PGR expression as a model to study advanced cancer stages. We showed that P4 treatment at clinically relevant doses stimulated cell proliferation in the DLD-1 and HT-29 cell lines, suggesting its potential role in the progression of colorectal cancer. DLD-1 and HT-29 cell lines have been widely used in various CRC studies [53,54]. The study by Tankiewicz-Kwedlo et al. demonstrated that the effects of Epo therapy on tumor growth dynamics were more pronounced in HT-29 cell xenografts compared to DLD-1 cell xenografts [55]. Sihong et al. suggested that HT-29 cells may have a low metastatic potential [56]. These variations in the behavior and response to treatment of DLD-1 and HT-29 cells may be attributed to their own distinct genetic profiles, microsatellite stability, potential mutations, gene expression patterns, and genomic alterations. This is of particular significance in cancer research, as it can aid in identifying potential drug targets and treatment strategies.

Similarly, P4 stimulation increased the proliferation of ovarian cancer cells in vitro and ovarian tumor growth in vivo through PGRMC1 [29]. In the present study, PGRMC1 inhibition abolished the effect of P4, suggesting that P4 in CRC may act primarily through PGRMC1. However, it is still uncertain whether PGRMC1 is an independent P4 receptor or requires additional P4-binding proteins for signaling. It has been suggested that PGRMC1 might act as a downstream mediator for other P4-binding proteins [10]. Possible binding partners for PGRMC1 include SERBP1 [10], microsomal cytochrome P450 (CYP) monooxygenase systems, and NENF [17,57]. The structural similarities between PGRMC1 and NENF have been demonstrated in previous studies [13]. Based on the fact that PGRMC1 is involved in the regulation of rapid non-genomic P4 actions, it has been hypothesized that NENF may also play a role in this type of P4 regulation [58]. Our results showed that PGRMC1 and NENF were co-expressed in the cytoplasm of the CRC cells, suggesting NENF as a potential cofactor for PGRMC1. Co-treatment with P4 and NENF increased the invasiveness of the CRC cells. This effect was abolished by a blockage of PGRMC1, indicating an important interaction between NENF and PGRMC1 for P4-mediated cell migration. Additionally, we showed that P4 increased the NENF secretion in the CRC cells, which suggests a direct effect of P4 on NENF production in colorectal cancer. 

The role of NENF in cancer biology, progression, or metastasis has not been extensively investigated [16,17], but it has been proposed that it may play a significant role in the development of liver, bladder, and breast cancers [16]. The depletion of NENF has been shown to reduce cancer cell growth and invasiveness and impair the ability of liver cancer cells to form tumors in mice [16]. NENF also increases the tumorigenicity and invasiveness of MCF-7 breast cancer cells [15]. Recently, we found that the concentration of NENF in the cerebrospinal fluid of patients with astrocytic brain tumors was significantly higher compared to that of non-tumoral individuals [17]. We also observed a strong correlation between the serum NENF concentration and its levels in cerebrospinal fluid, and noted that these levels were strongly gender-dependent [17]. Our present study showed an abundant NENF expression in the CRC tissues and a significantly higher serum concentration of NENF in patients with CRC compared to healthy controls. A diagnostic analysis revealed the usefulness of serum NENF levels in identifying CRC patients from those without cancer, suggesting its potential role as a circulating biomarker for colorectal cancer. 

Cancer cell proliferation and survival may be promoted by the activation of MAPK and PI3K/AKT signaling [13]. It has been shown that NENF and the pro-inflammatory cytokine IL-8 may also regulate these pathways [13,59]. However, the specific mechanism of IL-8 regulation in colorectal cancer requires further investigation. IL-8 is a versatile cytokine that has been shown to promote angiogenesis, attract immune cells, and stimulate tumor growth, invasion, and migration through both autocrine and paracrine effects [60,61,62]. Previous research has also shown that IL-8 can serve as a biomarker associated with a poor prognosis and chemoresistance for various types of cancer [63,64]. Additionally, IL-8 has been linked to adverse outcomes in brain tumors [65] and breast cancer [66]. In our study, we found that *IL-8* expression was significantly up-regulated in the CRC compared to the NM tissues. Moreover, NENF treatment increased the *IL-8* expression in both the DLD-1 and HT-29 cells, as well as the IL-8 release into the medium of the DLD-1 cells after P4 or NENF treatment without a PGRMC1 inhibitor (AG-205). This effect was abolished by PGRMC1 inhibition, suggesting that P4 and NENF require PGRMC1 to regulate IL-8 in colorectal cancer. Conversely, Emmanouil et al. suggested that HT-29 cells exhibit an increased metastatic potential and secrete angiogenic chemokines, notably IL-8 and VEGF, fostering neoangiogenesis and tumor advancement [67].

To perform *IL-8* knockdown, or even better, to knockout through CRISPR/CAS9 technology, experiments could elucidate the role of IL-8 in mediating the proliferation or invasion effects of P4 on CRC cells. This should be conducted in the future to enhance our knowledge on the mechanistic aspects of this. One limitation of our study was the absence of in vivo experiments, which could have provided additional support for our in vitro findings.

## 5. Conclusions

**Figure 7:**
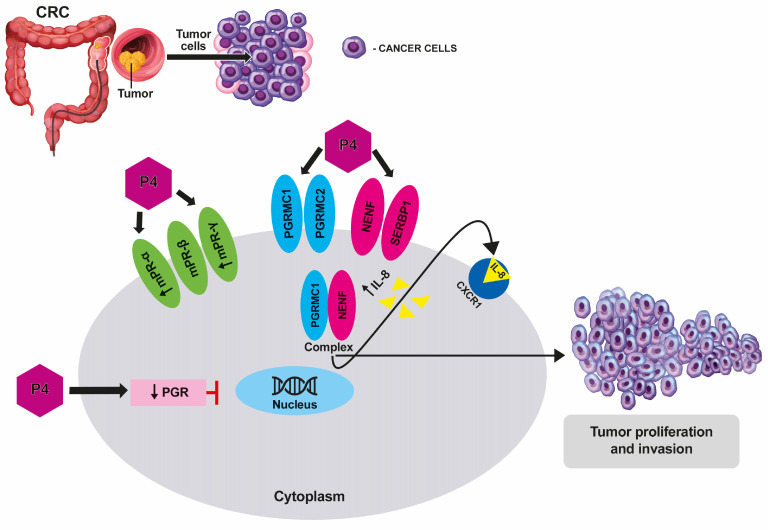
Schematic overview of the potential non-genomic P4 action in colorectal cancer. P4 may initiate rapid non-classical signaling through the complex of PGRMC1 and NENF, leading to increased proliferation and invasion of colorectal cancer cells. However, P4 cannot activate the classical genomic signaling pathway due to weak PGR expression in colorectal cancer cells (arrow: ↓*PGR*—weak PGR expression). P4 or NENF may significantly increase the release of IL-8 by colorectal cancer cells (arrow: ↑IL-8—increased release of IL-8). P4 significantly up-regulates *mPRα* and *mPRγ* expression in colorectal cancer cells (arrow: ↑*mPR-α*, ↑*mPR-γ*—increased expression of *mPR-α* and *mPR-γ*). NENF, neuron-derived neurotrophic factor; P4, progesterone; PGR, nuclear progesterone receptor; and PGRMC1, progesterone receptor membrane component 1.

Taken together, our data provided novel insights into the actions of P4, PGRMC1, and NENF in colorectal cancer, emphasizing new potential actions that may regulate CRC biology (summarized in Figure 7). In this action, it seems that PGRMC1 and NENF may interact as possible cofactors in non-classical P4 signaling. Targeting the PGRMC1/NENF complex may open-up new therapeutic possibilities for patients with colorectal cancer. Therefore, future studies aimed at developing treatment strategies for CRC could consider not only PGRMC1 inhibition, but also a blockage of NENF production and secretion. Moreover, NENF, as a secreted protein, could become a promising circulating biomarker candidate to distinguish between colorectal cancer patients and healthy individuals.

## Data Availability

The data generated in this study are available upon request from the corresponding author (V.D.-P., J.K.).

## Supplementary Materials

The following supporting information can be downloaded at: https://www.mdpi.com/article/10.3390/cancers15205074/s1. Table S1. Progesterone (P4) receptors characteristics regarding different clinicopathological parameters in colorectal cancer (CRC) patients. Table S2. Sequences of primers used in the q-RT-PCR. Table S3. Diagnostic usefulness of serum NENF evaluation in differentiating colorectal cancer patients from healthy individuals. Figure S1. Characterization of progesterone receptors and NENF expression profiles in DLD-1 and HT-29 cell lines. Figure S2. The area under the ROC curve (AUC) to distinguish between colorectal cancer patients and healthy individuals based on serum NENF levels.
