# Supplementary material for: New Insights on the Progesterone (P4) and PGRMC1/NENF Complex Interactions in Colorectal Cancer Progression
Source: Cancers (Basel). 2023 Oct 20;15(20):5074. doi: 10.3390/cancers15205074 (PMC10605590; doi:10.3390/cancers15205074)
Supplement: Supplementary file 1 [file cancers-15-05074-s001.zip › cancers-2578557-supplementary.pdf]

SUPPLEMENTAL DATA

Title: New insights on the progesterone (P4) and PGRMC1/NENF complex interactions in colorectal cancer progression

Table S1. Progesterone (P4) receptors characteristics regarding different clinicopathological parameters in colorectal cancer (CRC) patients.

Authors	Material/Method	Type of P4 receptor	Results
Kaklamanos et al., 1999	colorectal adenocarcinoma tissues (n=65)/IHC	PR	- PR expression was found in 23% (15/65) of CRC samples.<br>- PR expression was higher in women as compared to men.<br>- PR-positive expression was associated with a higher WHO grade.<br>- PR-positive expression was associated with a significantly shorter 5-year survival rate as compared to PR-negative samples.
Zavarhei et al., 2007	primary colorectal adenocarcinoma tissues and matched NM tissues (n=83)/IHC	PGR	- PGR expression was found in 59% (49/83) of CRC tissues and 15.6% (13/83) of NM tissues.<br>- PGR-negative expression was significantly associated with larger tumor size, higher Duke stage, and increased presence of secondary metastasis.
Qasim et al., 2011	colorectal adenoma tissues (n=33), colorectal adenocarcinoma tissues (n=33), non-tumorous colonic tissues (n=20)/IHC	PR	- PR expression gradually increased from normal mucosa (10%; 2/20) through adenoma (24.24%; 8/33) to adenocarcinoma (36.36%; 12/33).<br>- The 3-digital IHC parameters (A, N, I) were also significantly increasing from normal mucosa through adenoma to adenocarcinoma.
Liu, 2016	Datasets with the PR pathway activity data for colorectal adenoma, CRC, and normal colorectal mucosa (GSE20916, GSE37364, GSE17536, GSE39582, GSE14333, GSE17537, GSE33113, GSE12945, GSE44861)/gene signature-based BinReg approach	PR	- The PR pathway activity was significantly lower in CRC and adenoma tissues compared to normal colorectal mucosa.<br>- Higher PR pathway activity was significantly associated with lower WHO grade and longer disease-free survival.<br>- PR pathway activity was not associated with CRC location, tumor grade, microsatellite stability phenotype, and patient’s age.
Silverstein et al., 2019	WHO IV grade CRC tissues of women diagnosed during the peripartum period and matched NM tissues (n=5)/IHC	PR	- One CRC tissue showed weak (1%) nuclear PR expression.<br>- All normal tissues were PR-negative.
Ye et al., 2019	non-metastatic CRC tissues (n=148)/IHC	PR	- The score of PR expression was low in 78.4% (116/148) of CRC patients.<br>- PR expression was not associated with the patient's overall survival, local relapse-free survival, and distant metastasis-free survival.
ElLateef et al., 2020	colorectal adenocarcinoma tissues (n=30)/IHC	PR	- 76.67% (23/30) of CRC samples showed cytoplasmic PR expression from weak through moderate to strong. 23.33% (7/30) of CRC samples were PR-negative.<br>- PR-positive expression was significantly associated with higher cumulative progression-free survival.

			- PR expression was not related to the T stage, grades of the investigated tumors, and nodal metastasis.
Zhang et al., 2021	CRC tissues (n=77)/IHC	PGR	- PGR expression was high in 60% (49/77) of CRC tissues.<br>- High levels of PGR expression were associated with lower tumor size, tumor differentiation, vascular invasion, tumor stage, and longer short-term survival times.

Legend for Table S1: A, area; BinReg, Bayesian binary regression; CRC, colorectal cancer; DMFS, distant metastasis-free survival; I, intensity; IHC, immunohistochemical staining; n, number of cases; N, number of objects; NM, normal mucosa; PGR/PR, progesterone receptor; WHO, World Health Organization.

Table S2. Sequences of primers used in the q-RT-PCR.

Gene	Primer sequence (5'-3')	The product size (base pairs)	EMBL accesion number
*PPIA**	F:GCCAAGACTGAGTGGTTGGATG	144	NM_021130.4
	R:GAGTTGTCCACAGTCAGCAATGG		
PGR	F: GAGCACTGGATGCTGTTGCT	66	NM_001202474.3
	R: GGCTTAGGGCTTGGCTTTC		
mPRα	F: TGCCCTGCTGTGTGATCTTA	113	NM_178422.5
	R: ATAGCTGAGGCTCCTGGATG		
mPRβ	F: CGGTTGCATACCCTGTCCTG	152	NM_133367.4
	R: ATCTTGGGAAGCCCATCCTC		
mPRγ	F: ATTGTCCCAAGGCCTCAGAT	108	NM_001104554.1
	R: ATGCCATTCCAGTCAAATCC		
PGRMC1	F: TGCCTGGATAAGGAAGCACT	120	NM_006667.4
	R: GCCCACGTGATGATACTTGA		
PGRMC2	F: ATGGGAAAGTCTTCGACGTG	106	NM_006320.4
	R: CAAAATGTGGCCAGTCCTCT		
SERBP1	F: AGGACCGACAAGTCAAGTGC	106	NM_001018067.2
	R: CACAAAGGAACCAGGGTTGT		
NENF	F: AGGGGTAGCCAAGATGTCCT	149	NM_013349
	R: GAATTCTCCGGGCAGTGTAG		
CXCL8	F: ACCACACTGCGCCAACAC	101	NM_000584.4
	R: ACTTCTCCACAACCCTCTGC		
CXCR1	F: AGGTCCTGGGAAATGACACA	121	NM_000634.3
	R: AGTGTACGCAGGGTGAATCC		

* The reference gene (housekeeping gene)

Legend for Table S2: mPRα, membrane progesterone receptor alfa; mPRβ, membrane progesterone receptor beta; mPRγ, membrane progesterone receptor gamma; NENF, neudesin; PGR, classical progesterone receptor; PGRMC1, progesterone receptor membrane component 1; PGRMC2, progesterone receptor membrane component 2; PPIA, peptidylprolyl isomerase A; SERBP1, SERPINE1 mRNA binding protein.

Table S3. Diagnostic usefulness of serum NENF evaluation in differentiating colorectal cancer patients from healthy individuals.

	**Cut-off**	**Youden index**	**AUC ± SE**	**Se [%]**	**Sp [%]**	**PPV [%]**	**NPV [%]**	**ACC [%]**	**2-tailed *p*-value**
**NENF [ng/ml]**	2.97	0.30	0.676 ± 0.079	83	47	81	50	73	0.026

Legend for Table S3: ACC, diagnostic accuracy; AUC, the area under the ROC curve; Cut-off (based on the highest Youden index); NPV, negative predictive value; PPV, positive predictive value; SE, standard error; Se, diagnostic sensitivity; Sp, diagnostic specificity.

Figure S1

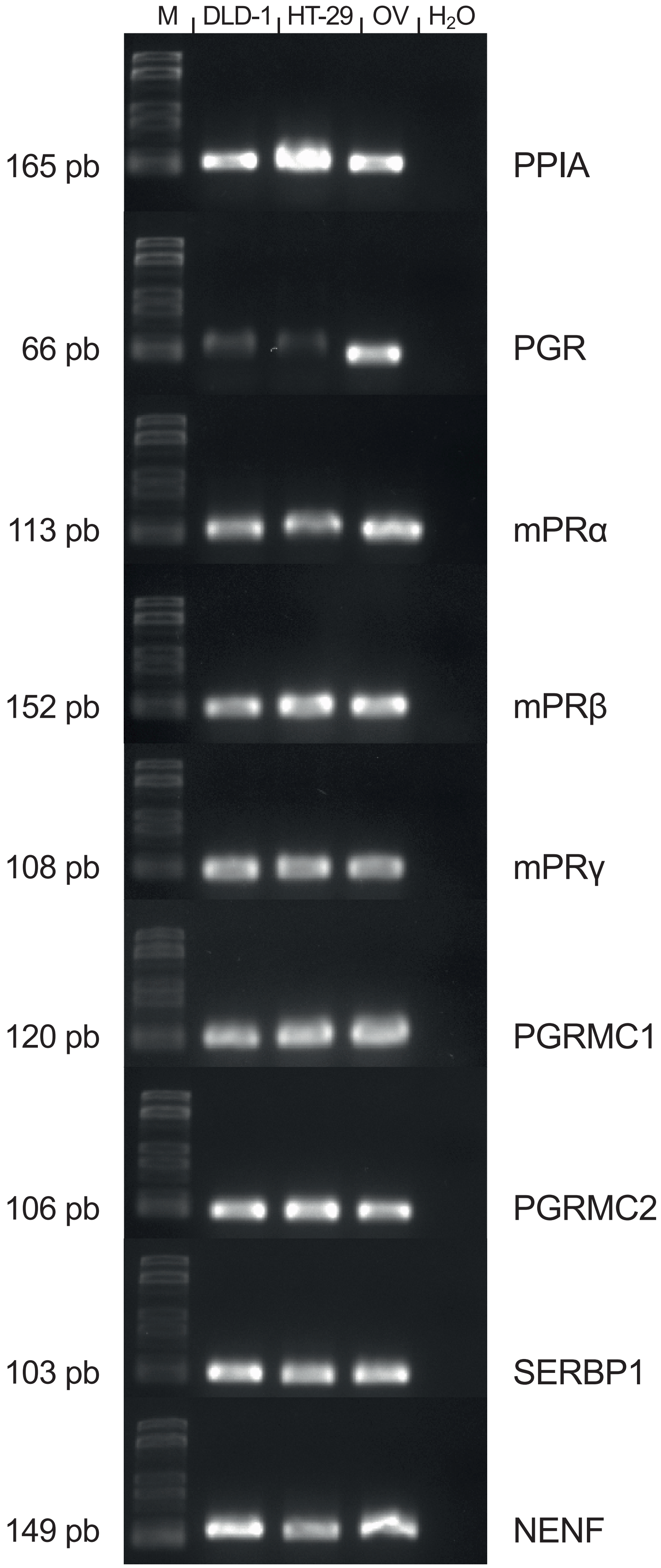
M
DLD-1
HT-29
OV
H_2O
165 pb
PPIA
66 pb
PGR
113 pb
mPRα
152 pb
mPRβ
108 pb
mPRγ
120 pb
PGRMC1
106 pb
PGRMC2
103 pb
SERBP1
149 pb
NENF

Figure S2

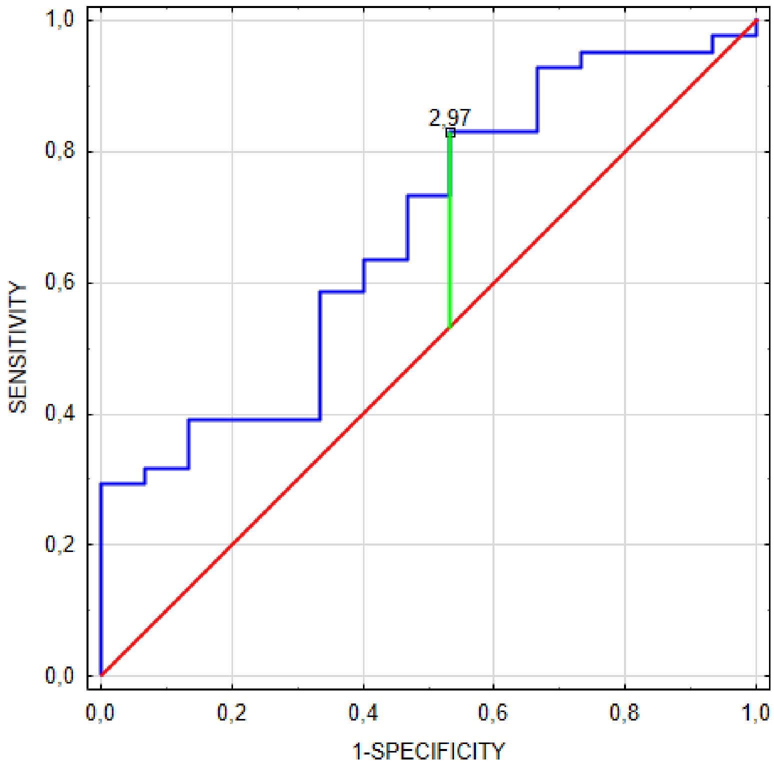

1,0
0,8
0,6
0,4
0,2
0,0
SENSITIVITY
2,97
0,0
0,2
0,4
0,6
0,8
1,0
1-SPECIFICITY

Figure S1. Characterization of progesterone receptors and NENF expression profiles in DLD-1 and HT-29 cell lines. The RT-PCR analysis in 2% agarose gel of housekeeping *PPIA*, PGR, mPRα, mPRβ, mPRγ, PGRMC1, PGRMC2, SERBP1 and *NENF* in DLD-1 and HT-29 cell lines. Amplicons' size is presented on the left. CRC, colorectal cancer; H_20, nuclease-free water; M, marker; mPRα, membrane progesterone receptor alfa; mPRβ, membrane progesterone receptor beta; mPRγ, membrane progesterone receptor gamma; NENF, neudesin; OV, ovary; P4, progesterone; PGR, classical progesterone receptor; PGRMC1, progesterone receptor membrane component 1; PGRMC2, progesterone receptor membrane component 2; PPIA, peptidylprolyl isomerase A; SERBP1, SERPINE1 mRNA binding protein.

Figure S2. The area under the ROC curve (AUC) to distinguish between colorectal cancer patients and healthy individuals based on serum NENF levels.

AUC = 0.676 for serum NENF; cut-off = 2.97 ng/ml.
